# Evaluating a Smartphone App (MeT4VeT) to Support the Mental Health of UK Armed Forces Veterans: Feasibility Randomized Controlled Trial

**DOI:** 10.2196/46508

**Published:** 2023-08-28

**Authors:** Steven Parkes, Bethany Croak, Samantha K Brooks, Sharon A M Stevelink, Daniel Leightley, Nicola T Fear, Laura Rafferty, Neil Greenberg

**Affiliations:** 1 King’s Centre for Military Health Research Institute of Psychiatry, Psychology & Neuroscience King’s College London London United Kingdom; 2 Department of Psychological Medicine Institute of Psychiatry, Psychology & Neuroscience King’s College London London United Kingdom; 3 Academic Department of Military Mental Health Institute of Psychiatry, Psychology & Neuroscience King’s College London London United Kingdom

**Keywords:** military, veteran, mental health, military to civilian transition, digital health, mobile apps, smartphone, mobile phone, mobile health, mHealth, digital intervention, support, app, feasibility, acceptability, engagement, usability

## Abstract

**Background:**

Previous research demonstrates that less than 50% of military veterans experiencing mental health difficulties seek formal support. Veterans often struggle to identify problems as mental health difficulties. In addition, they may fail to recognize the need for support before reaching a crisis point and face difficulties navigating care pathways to access support.

**Objective:**

A feasibility trial was conducted to assess a novel digital smartphone app (Mental Health Toolkit for Veterans Project [MeT4VeT]) for UK Armed Forces (UKAF) veterans experiencing mental health difficulties. The trial aimed to explore the feasibility and acceptability of trial procedures for a later randomized controlled trial (RCT) and to assess the acceptability of the MeT4VeT app.

**Methods:**

Participants were recruited at UK military medical centers, by advertising on social media, and through veteran third-sector organizations between February and November 2021, and assessed for eligibility (male, owned a smartphone, served at least 2 years in the UKAF, left the UKAF within the last 2 years, not undertaking formal mental health treatment). Eligible participants were assigned, on a 1:1 ratio, to either the intervention group (full app) or a control group (noninteractive app with signposting information). Three key objectives were determined a priori to assess the practicality of running an RCT including an assessment of recruitment and retention, evaluation of the technical app delivery and measurement processes, and acceptability and usability of the intervention.

**Results:**

In total, 791 individuals completed the participant information sheet, of which 261 (33%) were ineligible, 377 (48%) declined or were unable to be contacted for consent, and 103 (13%) did not download the app or complete the baseline measures. Of this, 50 participants completed baseline measures and were randomly assigned to the intervention group (n=24) or the control group (n=26). The trial was effective at enabling both the technical delivery of the intervention and collection of outcome measures, with improvements in mental health demonstrated for the intervention group from baseline to the 3-month follow-up. Recruitment and retention challenges were highlighted with only 50 out of the 530 eligible participants enrolled in the trial. The acceptability and usability of the MeT4VeT app were generally supported, and it was reported to be a useful, accessible way for veterans to monitor and manage their mental health.

**Conclusions:**

The results highlighted that further work is needed to refine recruitment processes and maintain engagement with the app. Following this, an RCT can be considered to robustly assess the ability of the app to positively affect mental health outcomes indicated within this trial.

**Trial Registration:**

ClinicalTrials.gov NCT05993676; https://clinicaltrials.gov/ct2/show/NCT05993676

## Introduction

Most of the 14,000 individuals who leave the UK Armed Forces (UKAF) every year [1] transition to civilian life successfully; however, some experience difficulties with their mental health [2]. Approximately 6% of UK veterans are likely to have posttraumatic stress disorder (PTSD), 22% report symptoms of common mental disorders such as anxiety or depression, and 10% report alcohol misuse [2]. More than half of the veterans experiencing mental health difficulties do not seek professional help [3], with studies consistently demonstrating that veterans often prefer to manage their mental health problems alone [4]. Veterans with mental health difficulties are prone to negative life outcomes, such as lower employment rates [5,6], emphasizing the need to encourage mental health support in this population.

Previous research investigating barriers to help-seeking in male veterans [7] identified 3 core barriers: veterans struggled to define the problems they were experiencing as mental health difficulties; veterans did not recognize their need for help until their difficulty reached a crisis point; and once help was sought, veterans found it challenging to navigate the care pathway and make sense of the number of services available to them. Given the poor uptake of formal mental health support in this population and the drive to manage mental health independently, an app that enables veterans to actively manage their symptoms and encourages them to access formal care if self-help has been ineffective could be of substantial benefit.

The Mental Health Toolkit for Veterans Project (MeT4VeT) was initiated to develop a smartphone app to help transitioning UK veterans by identifying mental health difficulties, providing a degree of self-help, and assisting them in recognizing when more formal support is required [8]. The app was developed by King’s Centre for Military Health Research, King’s College London according to the UK Medical Research Council (MRC) complex intervention framework [9,10]. This included a plan for the app development process, the involvement of veterans and stakeholders from services providing veteran support, and a scoping review of published research evidence for existing toolkits (eg, apps and websites). A smartphone app was the preferred format because of its geographical reach, 24/7 availability, lack of requirement for face-to-face contact, and ability to be downloaded at no cost [11]. The app was designed according to the MRC complex intervention framework by drawing on existing theories of cognitive behavioral therapy (CBT), which have been used as the foundation for many self-help tools and have demonstrated effectiveness in treating common mental disorders [12].

The MeT4VeT app consisted of five main sections: (1) *People*, enabling participants to identify symptoms of mental health difficulties through veterans’ stories; (2) *Tasks*, encouraging participants to set tasks to help them achieve goals in different areas of their life, for example, work, family and physical health; (3) *Tools*, providing participants with resources to independently manage their mental health; (4) *Tracking*, allowing participants to monitor their mental health and see their progression through the app; (5) *Notifications*, daily notifications to encourage engagement with the app. The elements of the app represent the intersection between core components of CBT, behavior change constructs, and potential means to overcome each of the barriers outlined in earlier research [7]. Core principles of CBT include the self-monitoring of thoughts, feelings, and behaviors (incorporated within the People section), behavioral activation and goal setting (included within the Tasks section), the use of techniques such as mindfulness and cognitive reframing (contained within the Tools section), and feedback on behavior (included in the Tracking section) [12].

This paper outlines a feasibility trial conducted in line with the MRC complex intervention framework [9,10]. The framework outlines the 4 phases in the development of an intervention and the trial focused on the second phase: “feasibility: assessing feasibility and acceptability of intervention and evaluation design to make decisions about progression to the next stage of evaluation” [10].

In line with this framework, a set of objectives were created to cover the following three core areas:

1. Assess the ability to recruit and retain participants throughout the trial period (28 days);
2. Evaluate technical app delivery and measurement processes, including the acceptability of 4 outcome measures: General Health Questionnaire (GHQ-12), PTSD Checklist-Civilian Version (PCL-C), Warwick Edinburgh Mental Well-Being Scale (WEMWBS), and World Health Organization Quality of Life Assessment (WHOQOL-BREF);
3. Examine the acceptability of the intervention, including engagement with and usability of the intervention.

## Methods

### Design

The trial had a parallel design. Participants were randomly assigned on a 1:1 ratio using block randomization to either an intervention group, who received the full smartphone app, or a control group, who received a noninteractive app that contained only signposting information. Data were collected at baseline and follow-up periods of 1 month (28 days) and 3 months (84 days) postbaseline. These time periods were chosen to align with other self-help intervention trials targeting a change in GHQ-12 scores and upon which our initial power calculation was based [13]. The time points enabled an assessment of any change at the end of the trial period (28 days) and again at 3 months to ascertain whether the change was continued. All participants were asked to use the app for a minimum of 28 days and app notifications were sent to the intervention group to encourage engagement.

### Participants

Participants were eligible for the study if they (1) are male, (2) own a smartphone, (3) served at least 2 years in the UKAF, (4) left the UKAF within the last 2 years, (5) are not currently undertaking formal mental health treatment (eg, mental health therapy conducted by a medical professional), and (6) indicated a degree of mental health distress assessed via a score of 2 or more on the GHQ-12 [14], similar to previous research [15]. Participants received compensation of a GBP £10 (US $12.85) Amazon voucher for completing each questionnaire (GBP £30 total, US $38.56), and participants in the intervention group who completed an interview received an additional GBP £10 (US $12.85) Amazon voucher.

### Recruitment

Participant recruitment was attempted at 3 UK military medical centers between October 2020 and February 2021. Personnel undergo a medical check-up when they leave the military as part of the discharge process, which includes a mental health screening. Three participating medical centers were asked to identify eligible participants at these check-ups and invite them to participate in the study. However, this approach became unfeasible due to the COVID-19 pandemic. The recruitment strategy was then expanded to include the use of social media and advertising in veteran third-sector organizations between February and November 2021. The social media platforms Facebook (Meta Platforms Inc), Instagram (Meta Platforms Inc), and Twitter (Twitter Inc) were used to post both paid and free promotional advertisements, which included a link to the participant information sheet and consent form on the questionnaire platform Qualtrics (Qualtrics International Inc).

### App Design

**Figure 1:**
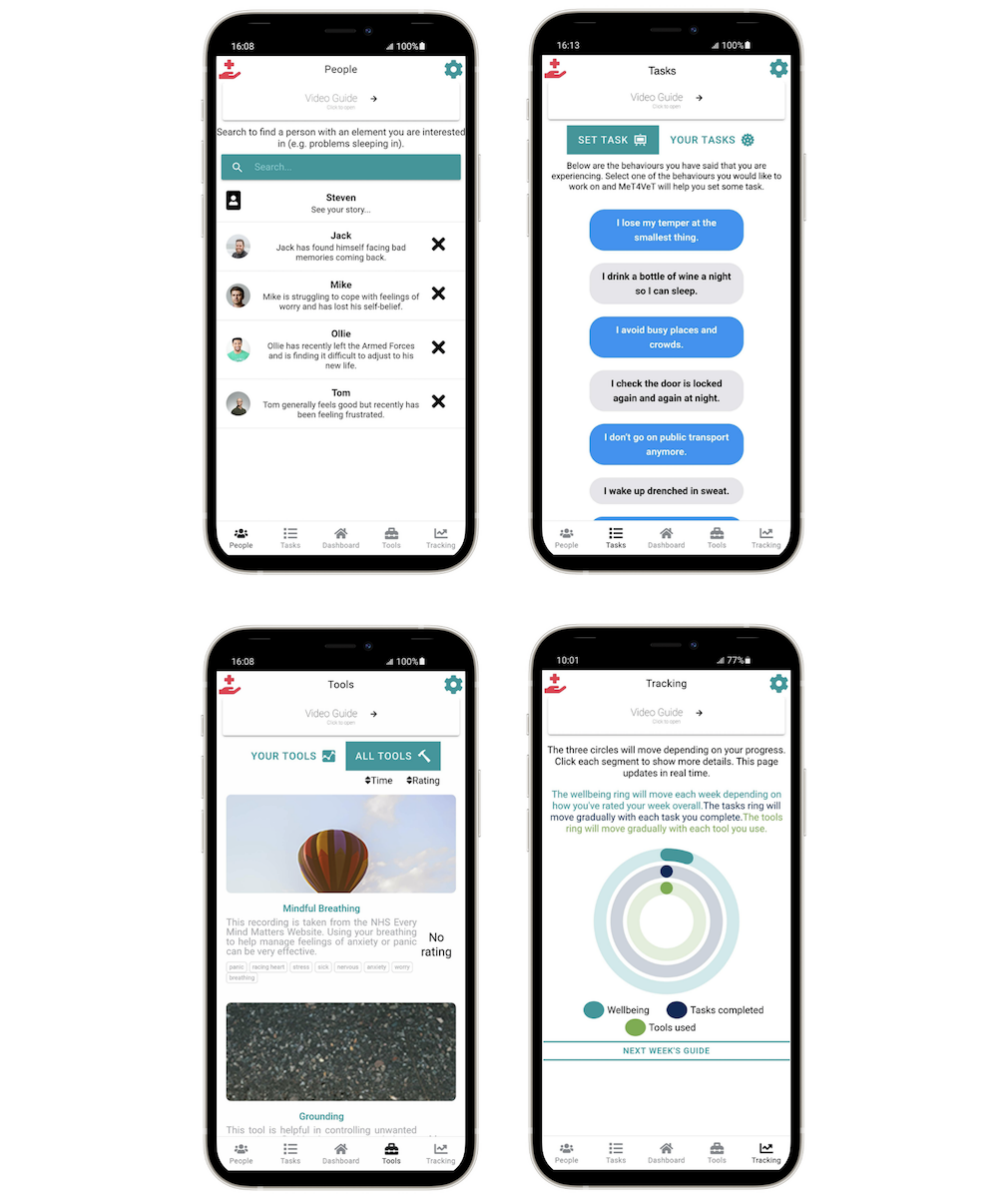
The Mental Health Toolkit for Veterans Project (MeT4VeT) app used by the intervention group.

Full details regarding the app development and its components have been reported previously [8]. The intervention group received the MeT4VeT app with full functionality (Figure 1 and Table 1). This included a daily schedule of notifications for the first 7 days related to the main app sections, followed by targeted notifications depending on participants’ usage of the app. For example, participants who completed at least 2 tasks in a week would receive the notification: “You’re making great progress with the ‘Tasks’ section. Are there any other behaviors you’d like to work on?” compared with participants who completed less than 2 tasks in a week: “It’s been a while since you have set some tasks, maybe add some more or change the ones you have set?” The control group received an app containing only signposting information about a range of statutory and third-sector organizations that focused on veterans’ mental health. Both apps were available on Android (Google) through the Google Play Store (Google) and on iOS (Apple Inc) via the TestFlight (Apple Inc) app.

**Table 1:** App sections and content for the intervention and control groups.

Accessed by and app section		Content	Intervention	Control
**First app launch and automatically displayed at follow-up points**				
	Questionnaires	Participants completed all questionnaires within the app after opening it for the first time and at subsequent follow-ups.	✓	✓
**Navigation menu**				
	Dashboard	After the questionnaires were completed, the dashboard was the main landing page. It contained information on how long the participant had been using the app, their name (set by the participant), and general advice for using the app.	✓	
	People	This section included 4 videos depicting fictional veterans discussing their mental health difficulties, corresponding to typical presentations of generalized anxiety disorder, depression, posttraumatic stress disorder, and a veteran who was struggling with mental health but did not necessarily meet a diagnostic threshold. After watching the videos, participants were asked to select the thoughts, feelings, and behaviors they related to in order to help them develop a picture of their mental health.	✓	
	Tasks	Participants were encouraged to identify areas of their lives that were important to them, such as work, family, or physical health, and to set a series of small tasks to help make changes in these areas.	✓	
	Tools	A range of mental health resources (eg, guided safe place imagery) were obtained from the National Health Service “Every Mind Matters” website [16]. These tools could either be accessed as a list or filtered to map onto the most appropriate ones to deal with the difficulties the veteran had selected in the earlier People section.	✓	
	Tracking	Participants were able to monitor their mental health through weekly check-ins, which were viewable in the Tracking section along with their progress across the app.	✓	
**Icons at the top of the app**				
	Signposting	A list of statutory and third-sector organizations that focused on veterans’ mental health.	✓	✓
	Settings	Information about the app, privacy policy, licenses, and questionnaire schedule. Participants were able to change their name, turn on or off notifications, and send feedback.	✓	✓
**Passive**				
	Notifications	Participants were encouraged to engage with the app through a schedule of daily notifications.	✓	

### Randomization

Once recruited into the study, participants were assigned a unique proxy identifier. This was linked to a randomization sequence generated in Stata (StataCorp) statistical software, which assigned participants on a 1:1 ratio to either the intervention or control group. All members of the research team were blind to group allocations except for authors SP, BC, and DL. SP and BC managed participant recruitment, conducted primary analyses, and had access to the raw study data. DL developed the MeT4VeT app and had access to the raw study data. Instructions for downloading the relevant app were sent to the participants via email. The apps could only be activated using a QR code or token code, which was sent to the participants by the research team upon successful registration to the trial.

### Trial Objectives

#### Overview

A set of objectives were developed a priori to assess areas of uncertainty before progression to a randomized controlled trial (RCT). These were based on the CONSORT (Consolidated Standards of Reporting Trials) guidelines for conducting a feasibility trial [17] (Table 2).

**Table 2:** Objectives to assess progression to a randomized controlled trial.

Criteria	Objectives
Recruitment and retention	1. Assess the number of eligible or interested participants. 2. Assess recruitment processes by calculating response rate. 3. Explore retention by estimating 1- and 3-month follow-up rates. 4. Determine the comparability of the sample to Ministry of Defence statistics for major subgroups of veterans (age, service, and rank).
Trial procedures and outcome measures	5. Evaluate the technical delivery of the app by gathering feedback from developers and participants. 6. Examine the feasibility of using the app to collect outcome measures by assessing completion rates and times. 7. Sample size estimation for the randomized controlled trial by assessing changes in mental health measures between groups.
Acceptability and usability	8. Explore the participants’ interaction with core areas of the app (People, Tasks, Tools, and Tracking) via app usage data. 9. Examine the participants’ interaction with the app throughout the trial period via app usage data. 10. Assess the usability of the app using the MAUQ^a^.

^a^MAUQ: mHealth App Usability Questionnaire.

#### Recruitment and Retention

Recruitment rates throughout the trial were presented according to the CONSORT flow diagram [17], with a breakdown of reasons provided for participant dropout. Demographic information was captured from all participants to assess their similarity to those who had left the UKAF in the last 2 years, drawn from UK Ministry of Defence (MOD) statistics [18]. Information was captured regarding age, military service branch, military rank, and regular or reservist status (Table 3).

**Table 3:** Summary of measures across all time points.

Measure		Baseline	Baseline + 1 month	Baseline + 3 months
**Demographics**		✓		
	Age			
	Ethnicity			
	Relationship status			
	Military service branch			
	Military rank			
	Regular or reservist status			
	Reason for leaving the military			
**App acceptability**			✓	
	Engagement: app usage data			
	Usability: MAUQ^a^			
**Outcome measures**		✓	✓	✓
	GHQ-12^b^			
	PCL-C^c^			
	WEMWBS^d^			
	WHOQOL-BREF^e^			

^a^MAUQ: mHealth App Usability Questionnaire.

^b^GHQ-12: General Health Questionnaire.

^c^PCL-C: PTSD Checklist-Civilian Version.

^d^WEMWBS: Warwick Edinburgh Mental Well-Being Scale.

^e^WHOQOL-BREF: World Health Organization Quality of Life Assessment.

#### Trial Procedures and Outcome Measures

Trial procedures were assessed by examining the technical delivery of the app and a review of the feedback option within the Settings section where participants were asked to note any technical issues as they occurred. The number of completed in-app surveys and the amount of time required to complete the surveys were also assessed.

Four outcome measures were trialed and completed by the participants in the Questionnaires section of the app. The GHQ-12 is a measure of common mental disorders such as anxiety and depression [14]. It is a 12-item self-report questionnaire measured on a 4-point scale. An example item is “Have you recently been feeling unhappy and depressed?” The GHQ-12 has demonstrated good psychometric properties (eg, Cronbach α=.91) [19].

To evaluate symptoms of PTSD, the PCL-C was used [20]. It is a 17-item self-report questionnaire, measured on a 5-point scale from (1) not at all to (5) extremely. An example item is “Over the past month how much have you been bothered by repeated, disturbing memories, thoughts or images of a stressful experience?” The PCL-C has been shown to have good psychometric properties (eg, Cronbach α=.96) [21].

The WEMWBS measures mental well-being within the previous 2 weeks and is a 14-item self-report questionnaire [22]. It is measured on a 5-point scale from (1) none of the time to (5) all of the time and an example item is “I’ve been thinking clearly.” The WEMWBS has been shown to have good internal consistency (Cronbach α=.89) and test-retest reliability (Cronbach α=.83) [22].

Quality of life was assessed using the WHOQOL-BREF, a 26-item self-report questionnaire [23]. It is measured on a 5-point scale and an example item is “How satisfied are you with yourself?” The WHOQOL-BREF has been shown to have good psychometric properties (eg, Cronbach α>.80 on 3 domains) [24].

#### Acceptability and Usability

App acceptability was assessed through engagement with the app, obtained from the app usage data. Engagement is defined as usage of the intervention including the temporal patterns (frequency and duration) and depth (specific intervention content) [25]. Engagement has been linked to behavior change through specific mechanisms of action such as the content and delivery of the intervention [25]. App usage data were collected from Google Analytics via Firebase and included information such as the number of times the app was opened, the duration of each app use, and the number of times specific sections of the app were used. Participants’ devices automatically reported the data to Google Analytics, although participants could choose to disable app tracking from their own devices outside of the MeT4VeT app. This type of automatic tracking of usage is a commonly used measure of engagement for digital behavior change interventions [25].

Usability data were obtained from the mHealth App Usability Questionnaire (MAUQ) [26], completed within the Questionnaires section of the app. The MAUQ is a 21-item self-report questionnaire measured on a 7-point scale ranging from strongly disagree (1) to strongly agree (7). Similar to a previous veteran app study [27], 5 questions were removed (resulting in a 16-item questionnaire), as the questions related to communicating with health care services through the app, which was not applicable in our study. Participant responses were aggregated into overall usability and 3 other domains: ease of use and satisfaction, interface and functionality, and usefulness [26]. The mean total score and the mean of each 3 domains were scored out of 7, with a score of 4 indicating a neutral response and higher scores indicating greater app usability. The MAUQ has been shown to have good psychometric properties (eg, Cronbach α>.80) for all 3 domains [26].

### Data Analysis

Data were analyzed using Stata v17 (StataCorp). For the outcome measures trialed, independent sample *t* tests and chi-square tests (for categorical data) were used to assess changes in outcome measures between the groups at 3 time points (baseline, baseline + 1 month, and baseline + 3 months) (Table S1 in Multimedia Appendix 1). App usage data were reported using medians and IQR. The MAUQ was reported using means and SD. A power analysis calculation was performed using the GHQ-12 with a threshold score of 3, as the common thresholds used in previous research are 2 or 3 [15]. Statistical significance is reported using *P* values with statistical significance being determined as *P*<.05.

### Ethics Approval

The feasibility trial was granted full ethical approval by the UK Ministry of Defence Research Ethics Committee (1074/MODREC/20) and the Research Governance Office at King’s College London (DPRF-19/20-16015).

## Results

### Overview

Three main areas of the feasibility trial were assessed (recruitment and retention; trial procedures and outcome measures; and acceptability and usability) to provide data on areas of uncertainty prior to progression to an RCT (Table 4).

**Table 4:** Objectives to assess progression to a randomized controlled trial and results from the Mental Health Toolkit for Veterans Project (MeT4VeT) feasibility trial.

Criteria and objectives			Results
**Recruitment and retention**			
	1. Assess the number of eligible or interested participants.	791 interested, 530 eligible (67%). Suggest opening criteria to women and those who have left the UK Armed Forces more than 2 years ago	
	2. Assess the recruitment processes by calculating the response rate.	Response rate of 29% (153/530) suggests refinements to minimize attrition	
	3. Explore retention by estimating 1- and 3-month follow-up rates.	Retention rates of 80% (40/50) at 1 month and 68% (34/50) at 3 months from baseline	
	4. Determine the comparability of the sample to UK MOD^a^ statistics for major subgroups of veterans (age, service, and rank).	Comparison conducted, suggest refinements to target younger veterans and women to enhance comparability	
**Trial procedures and outcome measures**			
	5. Evaluate the technical delivery of the app by gathering feedback from developers and participants.	Minor feedback resolved	
	6. Examine the feasibility of using the app to collect outcome measures by assessing completion rates and times.	Completion rates and times calculated and deemed acceptable by the research team	
	7. Sample size estimation for the randomized controlled trial by assessing changes in mental health measures between groups.	Power calculation conducted. Potential effectiveness of measures identified	
**Acceptability and usability**			
	8. Explore the participants’ interaction with core areas of the app (People, Tasks, Tools, and Tracking) via app usage data.	App usage data collected; suggest refinements to the Tools section of the app	
	9. Examine the participants’ interaction with the app throughout the trial period via app usage data.	App usage data collected; suggest refinements to maintain engagement	
	10. Assess the usability of the app using the MAUQ^b^.	Assessment conducted and deemed acceptable by the research team	

^a^MOD: Ministry of Defence.

^b^MAUQ: mHealth App Usability Questionnaire.

### Recruitment and Retention

**Figure 2:**
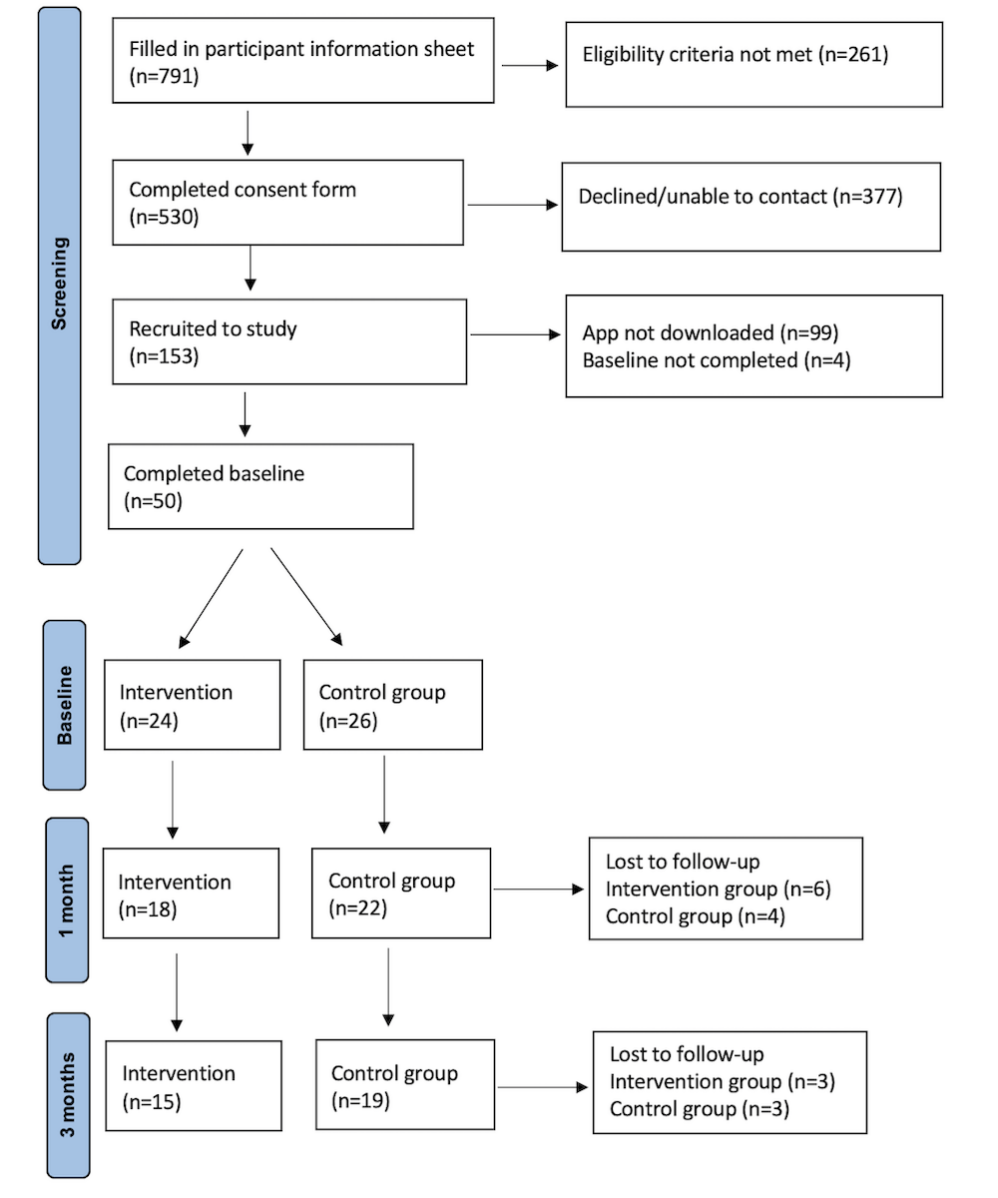
Participant recruitment flow diagram.

Following recruitment, 791 people completed the participant information sheet and 261 did not meet the eligibility criteria (Figure 2). There were multiple reasons why interested participants were not eligible to participate in the study (Table 5). The most common reasons were undergoing mental health treatment and not having left the UKAF in the last 2 years. A total of 530 participants completed the consent form and 377 of those either declined or could not be contacted for recruitment into the study. Overall, 153 participants were recruited into the study, but 99 did not download the app and 4 did not complete the baseline measures. The final sample consisted of 50 participants randomly assigned to the intervention group (n=24) and control group (n=26). After 1 month, 10 participants were lost to follow-up (n=6 intervention group, n=4 control group) and after 3 months, another 6 were lost to follow-up (n=3 in both groups).

**Table 5:** Reasons why participants were not eligible to participate in the trial.

Criteria^a^	Participants (n=261), n
Currently undertaking mental health treatment	110
Did not leave the UK Armed Forces in the last 2 years	86
Score of <2 on the GHQ-12^b^	74
Incomplete response on the participant information sheet	16
Did not serve in the UK Armed Forces for at least 2 years	9
Not male	5

^a^Some participants were ineligible because of multiple criteria.

^b^GHQ-12: General Health Questionnaire.

At baseline, there were no significant differences between the groups for any participant characteristic (Table 6). The mean age of the participants was 41.7 years (95% CI 38.8-44.7). Participants were predominantly White (47/50, 94%) and married, cohabiting, or in a long-term relationship (41/50, 82%). Two-thirds of participants had served in the Army (32/50, 64%), followed by the Royal Navy or Royal Air Force (18/50, 36%). Participants were predominately noncommissioned officers (40/50, 80%) and regular serving personnel (45/50, 90%). Of the reported reasons for leaving the military, there were several responses in the “other” category (25/50, 50%) such as health problems and dissatisfaction with their military career. Over half of all participants used an Android device (27/50, 54%).

**Table 6:** Participant characteristics, military background, and device information.

Characteristics			Total (N=50)^a^, n (%)		Intervention (n=24)^a^, n (%)		Control (n=26)^a^, n (%)
**Age (years)**							
	Younger than 40	18 (36)		10 (42)		8 (31)	
	40 to 49	19 (38)		8 (33)		11 (42)	
	50 and older	13 (26)		6 (25)		7 (27)	
**Relationship status**							
	In a relationship	41 (82)		20 (83)		21 (81)	
	Not in a relationship	9 (18)		4 (17)		5 (19)	
**Service branch**							
	Army	32 (64)		14 (58)		18 (69)	
	Royal Navy or Royal Air Force	18 (36)		10 (42)		8 (31)	
**Military rank**							
	Officer	10 (20)		4 (17)		6 (23)	
	Nonofficer	40 (80)		20 (83)		20 (77)	
**Reason for leaving the Armed Forces**							
	Completed term of service	17 (34)		9 (37)		8 (31)	
	Impact of service life on family	8 (16)		4 (17)		4 (15)	
	Other	25 (50)		11 (46)		14 (54)	
**Device operating system**							
	Android	27 (54)		14 (58)		13 (50)	
	iOS	23 (46)		10 (42)		13 (50)	

^a^When cells had less than 4 participants, categories were combined.

Participant characteristics were compared to the UK MOD personnel statistics [18] for male UKAF regulars leaving service from March 2020 to March 2022 (n=62,740). Statistics including reservists were not available by gender. The findings indicated an approximate mapping for rank: officer, 20% (11% MOD); other ranks, 80% (89% MOD); and service branch (Army, 64% [61% MOD], Royal Navy or Royal Air Force, 36% [39% MOD], Royal Navy or Royal Marines and Royal Air Force). Comparison of the age of participants highlights a far younger age demographic compared to the population included in this study: 36% (75% MOD) were younger than 40 years, 38% (17% MOD) were 40 to 49 years, and 26% (8% MOD) were 50 years and older.

A power calculation was performed to determine the sample size needed for a future RCT based on the difference in GHQ-12 scores at baseline and follow-up (3 months). To detect a difference in the GHQ-12 means of 3 points between groups at the 3-month follow-up, with 80% power and a significance level of 5%, each group would need to have 39 participants (n=78 in total). To allow for an attrition rate of 32% at the 3-month follow-up (16/50) based on this feasibility trial, 115 participants would need to be recruited. To account for a response rate of 29% (153/530) based on this feasibility trial, recruiting 397 participants is recommended for a future RCT.

### Trial Procedures and Outcome Measures

Technical delivery of the app was successful, with only minor resolvable technical issues identified by either the research team or the participants. Three participants (total N=50) provided technical feedback within the app; 1 needed support to clear notifications and 2 participants from the control group questioned whether there was additional content. The protocol was successful in collecting all measures outlined and the participants completed 124 surveys within the app, totaling 19 hours (median 21 minutes per participant).

At baseline, there were no significant differences between the groups on any outcome measures (Table S1 in Multimedia Appendix 1). For the intervention group, symptoms of mental health distress (GHQ-12) decreased significantly (*P*<.001) from preintervention (mean 7.0, SD 3.6) to the 3-month follow-up (mean 2.7, SD 3.6) with a mean difference of 4.3 (95% CI 1.9-6.7) (Table S4 in Multimedia Appendix 1). Symptoms of PTSD (PCL-C) decreased significantly (*P*=.03) from preintervention (mean 44.2, SD 15.0) to the 3-month follow-up (mean 33.2, SD 13.9) with a mean difference of 11.0 (95% CI 1.3-20.7). Well-being (WEMWBS) improved significantly (*P*=.04) from preintervention (mean 38.3, SD 9.2) to the 3-month follow-up (mean 44.9, SD 9.4) with a mean difference of −6.6 (95% CI −12.8 to −0.5). For the WHOQOL-BREF subdomain of psychological, quality of life increased significantly (*P*=.01) from preintervention (mean 11.1, SD 2.6) to the 3-month follow-up (mean 13.3, SD 2.4) with a mean difference of −2.2 (95% CI −3.9 to −0.5).

For the control group, there were no significant differences in any of the outcome measures from preintervention to the 3-month follow-up (Table S5 in Multimedia Appendix 1). Analyses of the outcome measures across all time points are available in Multimedia Appendix 1.

### Acceptability

Over the trial period (28 days), the intervention group initialized the app a median of 8.5 times (IQR 3-18) and over a median period of 2.5 weeks (IQR 1-3.5) (Table 7). The median session duration was 20.7 seconds (IQR 17.2-34.3). The control group initialized the noninteractive app a median of 2 times (IQR 1-6) and over a median period of 1 week (IQR 1-1). The median session duration was 29 seconds (IQR 20-39.2).

**Table 7:** App usage per participant for both groups over the trial period (28 days).

App usage	Intervention (n=24), median (IQR)	Control (n=26), median (IQR)
Initializations^a^	8.5 (3-18)	2 (1-6)
Session count^b^	52 (12-112)	15.5 (7-27)
Session duration (seconds)	20.7 (17.2-34.3)	29 (20-39.2)
Server interactions^c^	9.5 (4-40.5)	5 (4-8)
Weeks active	2.5 (1-3.5)	1 (1-1)

^a^Number of times the app was opened as a new instance.

^b^Number of times the app was reopened after running in the background.

^c^Number of times the app interacted with the server.

The People section of the app was accessed most frequently by the intervention group with an average of 14 (IQR 4-45) visits per participant, followed by the Dashboard section with an average of 11 (IQR 6-21) visits per participant (Table 8). Other than the Questionnaires section, participants spent the most time on the People section with an average visit length of 2.8 minutes (median 169.3 seconds, IQR 54.2-430.4). Out of the 4 main sections of the app (People, Tasks, Tools, and Tracking), the Tracking section was accessed by the least number of participants (n=19, 79%), the Tasks section was used the least number of times (median 8.5, IQR 4-43) and the Tools section was used for the least amount of time (median 49.5, IQR 6.7-166.8).

**Table 8:** App usage for each section of the Mental Health Toolkit for Veterans Project (MeT4VeT) app in the intervention group over the 28-day trial period (n=24).

App screen	Ever accessed^a^, n (%)	Number of times accessed per person, median (IQR)	Time on screen per person (seconds), median (IQR)
Questionnaires^b^	23 (96)	2 (1-3)	552.5 (280.7-725.1)
Dashboard	23 (96)	11 (6-21)	103.3 (62.2-139.2)
People	22 (92)	14 (4-45)	169.3 (54.2-430.4)
Tasks	22 (92)	8.5 (4-43)	107.6 (25.5-452.8)
Tools	20 (83)	9 (2.5-23)	49.5 (6.7-166.8)
Tracking	19 (79)	9 (4-21)	68.9 (15.1-150.8)
Settings	16 (67)	3 (1.5-4)	14.9 (0-22.8)
Signposting	15 (63)	2 (2-6)	9.9 (0-40.8)

^a^During the trial, Apple (developer of iOS) changed policies related to how developers could track and monitor the usage of an app. This required specific user content, which could be modified outside the app. It is therefore not possible to ascertain whether a user did not provide data because they were not using the app or whether they declined to share app usage statistics.

^b^One participant accessed the app but did not open the Questionnaires screen until day 29.

### Usability

Participants in the intervention group rated the overall usability (mean 5.3, SD 1.5) on the MAUQ and each of the subdomains of ease of use and satisfaction (mean 5.5, SD 1.5), interface and functionality (mean 4.9, SD 1.6), and usefulness (mean 5.3, SD 1.8) of the app with positive (above neutral) responses. The control group had higher scores compared to the intervention group for overall usability (mean 5.6, SD 1.2), ease of use and satisfaction (mean 5.9, SD 1.1), interface and functionality (mean 5.5, SD 1.4), and lower scores for usefulness (mean 4.8, SD 1.6).

## Discussion

### Principal Findings

The aims of the trial were to collect data to determine the feasibility of running an RCT by assessing (1) recruitment and retention, (2) trial procedures and outcome measures, and (3) the acceptability of the MeT4VeT app.

### Recruitment and Retention

The COVID-19 pandemic prevented effective recruitment through military bases and social media was used as an alternative recruitment strategy. This presented some challenges in obtaining valid responses and retaining participants, which likely accounts for the high dropout rate. These challenges are common in research using social media as a recruitment tool [28,29]. If a future RCT uses social media recruitment, further work will be required to reduce dropout rates or increase the provision of resources to support this degree of dropout. Recruitment may have also been impacted by stringent restrictions from Apple for publishing apps on their app store and another app (TestFlight) was required to be installed before MeT4VeT could be downloaded, which may have led to reduced engagement. Once participants were engaged in the trial, the retention rate was 68% (34/50) from baseline measures to follow-up, which is acceptable compared to other trials assessing interventions within a military veteran population [30,31]. A trial exploring a mental health monitoring app within this population indicated a retention rate of 81% from baseline to follow-up [30] and a trial evaluating an alcohol use reduction intervention reported a retention rate of 22% to follow-up [31].

The sample in this study is comparable to UK MOD statistics [18] in terms of service branch and rank division across the UKAF. However, our sample represented a substantially older demographic when compared to those leaving the UKAF between 2020 and 2022 [18]. To improve comparability, we suggest recruiting from a wider age range to include younger veterans and females.

Examination of the eligibility criteria showed that a large number of potential participants were not eligible as they had not left the UKAF within the last 2 years. Future studies could relax this criterion to enable veterans to enter the trial at any stage of transition. An even greater number of potential participants were not eligible to participate because they were currently undertaking mental health treatment, which could indicate a need for the development of an app to enable self-management to complement therapy within this population. The presence of a group of potential participants who did not meet the GHQ-12 cut-off but were interested in an app to manage their mental health suggests a potentially broader scope of interest to maintain positive mental health or prevent mental health distress.

### Trial Procedures and Outcome Measures

The results of the trial indicate it would be feasible to conduct an RCT using the same technical delivery of the app and the same set of measurement processes. Four outcome measures were deemed acceptable for data collection, with the intervention indicating a potential improvement in mental health from preintervention to the 3-month follow-up, contrasting with no significant improvement in the GHQ-12 scores for the control group over the same time period. Due to the small sample size, it is difficult to draw any firm conclusions from these results, but they provide a promising signal for the MeT4VeT app and the use of the GHQ-12 as an outcome measure to be explored in a future RCT with longer follow-up periods and a larger sample size. A provisional sample size for a later RCT of 397 was calculated.

### Acceptability and Usability

The MAUQ scores indicated positive responses to the app, with scores ranging from 4.9 to 5.5, all of which were above a neutral response of 4. However, the scores were below those of a similar app designed to reduce veterans’ alcohol consumption where scores of 5.7 to 5.9 were reported across the MAUQ domains [31]. Before a future RCT, work should be conducted using qualitative feedback on the app to refine its usability. Qualitative data were collected from this trial and will be reported in a future publication.

The app usage data indicated that the intervention group engaged with the MeT4VeT app more often (initializations and sessions) and for a longer duration than the control group (weeks active). When focusing on the 4 core sections of the app (People, Tasks, Tools, and Tracking), the People section was accessed most frequently and for the longest duration and it required active participation. The remaining sections were accessed less frequently by participants. This may have been because they could be accessed via other resources (eg, Tools), whereas the People section provided a novel resource (videos with veterans) not readily available elsewhere. A future iteration of the app may be best focused as a gateway into existing self-help tools, with continued development of the People, Tasks, and Tracking sections and replacement of the Tools section with links to existing resources. This aligns with the original intention of the app, to create a way to help people at the beginning of their journey to identify symptoms and track them over time to recognize when difficulties occur and when self-help, or more formal support, may be beneficial. The median duration of app usage in the intervention group was 2.5 weeks, which falls below the 4-week trial period. Before an RCT is initiated, further research is required to explore the reasons behind early disengagement with the app and potentially refine the notification schedule to encourage app usage throughout the full trial period.

### Strengths

The strengths of this study include its rigorous design, with random assignment to groups and the inclusion of a control group. The MeT4VeT app development used a strong conceptual framework informed by the military mental health literature and previous research [7]. Furthermore, feedback on the app was provided throughout the app development process by stakeholders working on veterans’ mental health at 4 stakeholder events, through interviews with veterans, and veteran involvement and engagement groups.

### Limitations

The limitations of the study include the relatively small sample size, partly due to the COVID-19 pandemic, which impacted recruitment, and partly due to the loss of potential participants lacking interest in trial initiation. The generalizability of the results is affected by the limitations of the sample, which included only male veterans and those that had left the UKAF within the last 2 years. The development of the app was based on data drawn from research on male veterans [7], so the same barriers could not be assumed to be synonymous for the female veteran population. Future research should explore whether female veterans experience the same barriers and evaluate the use of the app in this population. The feasibility study tentatively demonstrated that MeT4VeT may be useful for those within 2 years of leaving the UKAF and whose departure from the UKAF was over a longer time period; further research is warranted to explore this.

### Conclusions

The results of the trial suggest that although the study protocol was both feasible and acceptable, the recruitment process requires further refinement to provide an appropriate number of participants for a future RCT. The outcome measures used within the trial were acceptable for use in a future RCT to robustly examine the effectiveness of the app. The findings indicate that the MeT4VeT app shows some promise as a useful, accessible way for veterans to monitor and manage their mental health.

## Appendix

Multimedia Appendix 1 Outcome measures for all time points.

Multimedia Appendix 2 CONSORT-eHEALTH checklist V 1.6.1.

## Abbreviations

CBT: cognitive behavioral therapy
CONSORT: Consolidated Standards of Reporting Trials
GHQ-12: General Health Questionnaire
MAUQ: mHealth App Usability Questionnaire
MeT4VeT: Mental Health Toolkit for Veterans Project
MOD: Ministry of Defence
MRC: Medical Research Council
PCL-C: PTSD Checklist-Civilian Version
PTSD: posttraumatic stress disorder
RCT: randomized controlled trial
UKAF: UK Armed Forces
WEMWBS: Warwick Edinburgh Mental Well-Being Scale
WHOQOL-BREF: World Health Organization Quality of Life Assessment

## References

[1] (2022). UK Armed Forces quarterly service personnel statistics. Ministry of Defence.

[2] Stevelink SAM, Jones M, Hull L, Pernet D, MacCrimmon S, Goodwin L, MacManus D, Murphy D, Jones N, Greenberg N, Rona RJ, Fear NT, Wessely S (2018). Mental health outcomes at the end of the British involvement in the Iraq and Afghanistan conflicts: a cohort study. Br J Psychiatry.

[3] Stevelink SAM, Jones N, Jones M, Dyball D, Khera CK, Pernet D, MacCrimmon S, Murphy D, Hull L, Greenberg N, MacManus D, Goodwin L, Sharp ML, Wessely S, Rona RJ, Fear NT (2019). Do serving and ex-serving personnel of the UK armed forces seek help for perceived stress, emotional or mental health problems?. Eur J Psychotraumatol.

[4] Shepardson RL, Tapio J, Funderburk JS (2017). Self-management strategies for stress and anxiety used by nontreatment seeking veteran primary care patients. Mil Med.

[5] Iversen A, Nikolaou V, Greenberg N, Unwin C, Hull L, Hotopf M, Dandeker C, Ross J, Wessely S (2005). What happens to British veterans when they leave the armed forces?. Eur J Public Health.

[6] Murphy D, Palmer E, Busuttil W (2017). Exploring indices of multiple deprivation within a sample of veterans seeking help for mental health difficulties residing in England. J Epidemiol Public Health Rev.

[7] Rafferty LA, Wessely S, Stevelink SAM, Greenberg N (2020). The journey to professional mental health support: a qualitative exploration of the barriers and facilitators impacting military veterans' engagement with mental health treatment. Eur J Psychotraumatol.

[8] Parkes S, Croak B, Brooks SK, Stevelink SAM, Fear NT, Rafferty L, Greenberg N (2022). MeT4VeT: development and assessment of a mental health toolkit for military veterans. Forces in Mind Trust.

[9] O'Cathain A, Croot L, Duncan E, Rousseau N, Sworn K, Turner KM, Yardley L, Hoddinott P (2019). Guidance on how to develop complex interventions to improve health and healthcare. BMJ Open.

[10] Skivington K, Matthews L, Simpson SA, Craig P, Baird J, Blazeby JM, Boyd KA, Craig N, French DP, McIntosh E, Petticrew M, Rycroft-Malone J, White M, Moore L (2021). A new framework for developing and evaluating complex interventions: update of Medical Research Council guidance. BMJ.

[11] Owen JE, Kuhn E, Jaworski BK, McGee-Vincent P, Juhasz K, Hoffman JE, Rosen C (2018). VA mobile apps for PTSD and related problems: public health resources for veterans and those who care for them. mHealth.

[12] Bakker D, Kazantzis N, Rickwood D, Rickard N (2016). Mental health smartphone apps: review and evidence-based recommendations for future developments. JMIR Ment Health.

[13] Bower P, Richards D, Lovell K (2001). The clinical and cost-effectiveness of self-help treatments for anxiety and depressive disorders in primary care: a systematic review. Br J Gen Pract.

[14] Goldberg DP, Hillier VF (1979). A scaled version of the General Health Questionnaire. Psychol Med.

[15] Goldberg DP, Gater R, Sartorius N, Ustun TB, Piccinelli M, Gureje O, Rutter C (1997). The validity of two versions of the GHQ in the WHO study of mental illness in general health care. Psychol Med.

[16] Every Mind Matters. National Health Service.

[17] Eldridge SM, Chan CL, Campbell MJ, Bond CM, Hopewell S, Thabane L, Lancaster GA, PAFS consensus group (2016). CONSORT 2010 statement: extension to randomised pilot and feasibility trials. BMJ.

[18] (2022). UK Armed Forces biannual diversity statistics: April 2022. Ministry of Defence.

[19] McCabe CJ, Thomas KJ, Brazier JE, Coleman P (1996). Measuring the mental health status of a population: a comparison of the GHQ-12 and the SF-36 (MHI-5). Br J Psychiatry.

[20] Weathers F, Litz B, Herman D, Huska J, Keane T (1993). The PTSD Checklist (PCL): reliability, validity, and diagnostic utility.

[21] Keen SM, Kutter CJ, Niles BL, Krinsley KE (2008). Psychometric properties of PTSD checklist in sample of male veterans. J Rehabil Res Dev.

[22] Tennant R, Hiller L, Fishwick R, Platt S, Joseph S, Weich S, Parkinson J, Secker J, Stewart-Brown S (2007). The Warwick-Edinburgh Mental Well-Being Scale (WEMWBS): development and UK validation. Health Qual Life Outcomes.

[23] The WHOQOL Group (1998). Development of the World Health Organization WHOQOL-BREF quality of life assessment. Psychol Med.

[24] Skevington SM, Lotfy M, O'Connell KA, WHOQOL Group (2004). The World Health Organization's WHOQOL-BREF quality of life assessment: psychometric properties and results of the international field trial. A report from the WHOQOL group. Qual Life Res.

[25] Perski O, Blandford A, West R, Michie S (2017). Conceptualising engagement with digital behaviour change interventions: a systematic review using principles from critical interpretive synthesis. Transl Behav Med.

[26] Zhou L, Bao J, Setiawan IMA, Saptono A, Parmanto B (2019). The mHealth App Usability Questionnaire (MAUQ): development and validation study. JMIR Mhealth Uhealth.

[27] Williamson C, Dryden D, Palmer L, Rona R, Simms A, Fear NT, Goodwin L, Murphy D, Leightley D (2022). An expert and veteran user assessment of the usability of an alcohol reduction app for military veterans, drinks:ration: a mixed-methods pilot study. Mil Behav Health.

[28] Williamson C, Rona RJ, Simms A, Fear NT, Goodwin L, Murphy D, Leightley D (2023). Recruiting military veterans into alcohol misuse research: the role of social media and Facebook advertising. Telemed J E Health.

[29] Lee K, Kwon H, Lee B, Lee G, Lee JH, Park YR, Shin SY (2018). Effect of self-monitoring on long-term patient engagement with mobile health applications. PLoS One.

[30] Betthauser LM, Stearns-Yoder KA, McGarity S, Smith V, Place S, Brenner LA (2020). Mobile app for mental health monitoring and clinical outreach in veterans: mixed methods feasibility and acceptability study. J Med Internet Res.

[31] Leightley D, Williamson C, Rona RJ, Carr E, Shearer J, Davis JP, Simms A, Fear NT, Goodwin L, Murphy D (2022). Evaluating the efficacy of the drinks:ration mobile app to reduce alcohol consumption in a help-seeking military veteran population: randomized controlled trial. JMIR Mhealth Uhealth.

