# Supplementary material for: Evaluating a Smartphone App (MeT4VeT) to Support the Mental Health of UK Armed Forces Veterans: Feasibility Randomized Controlled Trial
Source: JMIR Ment Health. 2023 Aug 28;10:e46508. doi: 10.2196/46508 (PMC10495851; doi:10.2196/46508)
Supplement: Multimedia Appendix 1 [file mental_v10i1e46508_app1.docx]

**Table S1**. Outcome measures and differences at each time point for total sample and by group.

| **Baseline** | **Measure** | **Total** | | **Intervention** | | **Control** | | **T-test (between groups)** |
| --- | --- | --- | --- | --- | --- | --- | --- | --- |
|  |  | *N* | Mean (SD) | *N* | Mean (SD) | *N* | Mean (SD) | *t*(*df*), *P* value |
|  | Mental health distress (GHQ-12) | 50 | 7.26 (3.69) | 24 | 6.96 (3.64) | 26 | 7.54 (3.79) | -0.55(48), .58 |
|  | PTSD symptoms (PCL-C) | 50 | 45.42 (14.62) | 24 | 44.17 (14.95) | 26 | 46.58 (14.50) | -0.58(48), .57 |
|  | Well-being (WEMWBS) | 50 | 37.66 (8.99) | 24 | 38.25 (9.15) | 26 | 37.12 (8.98) | 0.44(48), .67 |
|  | Quality of life (WHOQOL-BREF)  Environment | 50 | 13.22 (2.43) | 24 | 12.98 (2.40) | 26 | 13.44 (2.48) | -0.67(48), .51 |
|  | Physical health | 50 | 13.30 (2.65) | 24 | 13.05 (2.01) | 26 | 13.54 (3.16) | -0.65(48), .52 |
|  | Psychological | 50 | 11.2 (2.70) | 24 | 11.11 (2.60) | 26 | 11.28 (2.84) | -0.22(48), .83 |
|  | Social | 50 | 11.17 (3.79) | 24 | 10.89 (3.60) | 26 | 11.44 (4.01) | -0.51(48), .62 |
| **Baseline + 1 month** | Mental health distress (GHQ-12) | 40 | 3.4 (3.52) | 18 | 2.83 (3.49) | 22 | 3.86 (3.56) | -0.92(38), .36 |
|  | PTSD symptoms (PCL-C) | 40 | 37.83 (12.46) | 18 | 35.61 (11.48) | 22 | 39.64 (13.19) | -1.02(38), .32 |
|  | Well-being (WEMWBS) | 40 | 39.78 (9.78) | 18 | 41.11 (9.79) | 22 | 38.68 (9.87) | 0.78(38), .44 |
|  | Quality of life (WHOQOL-BREF)  Environment | 40 | 13.13 (2.71) | 18 | 13.22 (2.52) | 22 | 13.05 (2.91) | 0.20(38), .84 |
|  | Physical health | 40 | 13.69 (2.42) | 18 | 13.87 (2.24) | 22 | 13.53 (2.61) | 0.44(38), .66 |
|  | Psychological | 40 | 11.58 (2.93) | 18 | 12.41 (2.98) | 22 | 10.91 (2.78) | 1.64(38), .11 |
|  | Social | 40 | 11.9 (3.76) | 18 | 12.07 (4.10) | 22 | 11.76 (3.55) | 0.26(38), .80 |
| **Baseline + 3 months** | Mental health distress (GHQ-12) | 34 | 4.12 (4.22) | 15 | 2.67 (3.60) | 19 | 5.26 (4.41) | -1.85(32), .07 |
|  | PTSD symptoms (PCL-C) | 34 | 38.62 (15.84) | 15 | 33.2 (13.94) | 19 | 42.89 (16.29) | -1.83(32), .08 |
|  | Well-being (WEMWBS) | 34 | 41.59 (9.15) | 15 | 44.87 (9.36) | 19 | 39 (8.32) | 1.93(32), .06 |
|  | Quality of life (WHOQOL-BREF)  Environment | 34 | 13.12 (2.59) | 15 | 14.03 (2.43) | 19 | 12.39 (2.54) | 1.90(32), .07 |
|  | Physical health | 34 | 13.46 (3.22) | 15 | 13.87 (3.41) | 19 | 13.14 (3.12) | 0.64(32), .52 |
|  | Psychological | 34 | 12.22 (2.89) | 15 | 13.33 (2.40) | 19 | 11.33 (2.99) | **2.11(32), .04** |
|  | Social | 34 | 11.96 (3.79) | 15 | 13.24 (3.47) | 19 | 10.95 (3.81) | 1.81(32), .08 |

GHQ-12, General Health Questionnaire; PCL-C, PTSD Checklist Civilian version; WEMWBS, Warwick Edinburgh Mental Well-being Scale; WHOQOL-BREF World Health Organization Quality of Life Assessment. **Bold** indicates *P*<.05.

**Table S2**. Differences in outcome measures from pre-intervention to post-intervention and post-intervention to follow-up for the intervention group.

| **Measure** | **Pre-intervention (n=24) to post-intervention (n=18)** | | | | **Post-intervention (n=18) to follow-up (n=15)** | | | |
| --- | --- | --- | --- | --- | --- | --- | --- | --- |
|  | Baseline | Baseline +  1 month | Difference | T-test | Baseline +  1 month | Baseline +  3 months | Difference | T-test |
|  | Mean (SD) | Mean (SD) | Mean (95% CI) | *t*(*df*), *P* value | Mean (SD) | Mean (SD) | Mean (95% CI) | *t*(*df*), *P* value |
| Mental health distress (GHQ-12) | 6.96 (3.64) | 2.83 (3.49) | 4.13 (1.87, 6.38) | **3.70(40), <.001** | 2.83 (3.49) | 2.67 (3.60) | 0.17 (-2.36, 2.69) | 0.13(31), .89 |
| PTSD symptoms (PCL-C) | 44.17 (14.95) | 35.61 (11.48) | 8.56 (-0.00, 17.12) | 2.02(40), .05 | 35.61 (11.48) | 33.2 (13.94) | 2.41 (-6.61. 11.43) | 0.55(31), .59 |
| Well-being (WEMWBS) | 38.25 (9.15) | 41.11 (9.79) | -2.86 (-8.80, 3.08) | -0.97(40), .34 | 41.11 (9.79) | 44.87 (9.36) | -3.76 (-10.60, 3.09) | -1.12(31), .27 |
| Quality of life (WHOQOL-BREF)  Environment | 12.98 (2.40) | 13.22 (2.52) | -0.24 (-1.79, 1.30) | -0.32(40), .76 | 13.22 (2.52) | 14.03 (2.43) | -0.81 (-2.58, 0.96) | -0.94(31), .36 |
| Physical health | 13.05 (2.01) | 13.87 (2.24) | -0.83 (-2.15, 0.50) | -1.26(40), .22 | 13.87 (2.24) | 13.87 (3.41) | 0.01 (-2.01, 2.02) | 0.01(31), .99 |
| Psychological | 11.11 (2.60) | 12.41 (2.98) | -1.30 (-3.04, 0.45) | -1.50(40), .14 | 12.41 (2.98) | 13.33 (2.40) | -0.93 (-2.88, 1.03) | -0.97(31), .34 |
| Social | 10.89 (3.60) | 12.07 (4.10) | -1.19 (-3.59, 1.22) | -0.99(40), .33 | 12.07 (4.10) | 13.24 (3.47) | -1.17 (-3.90, 1.56) | -0.87(31), .39 |

GHQ-12, General Health Questionnaire; PCL-C, PTSD Checklist Civilian version; WEMWBS, Warwick Edinburgh Mental Well-being Scale; WHOQOL-BREF World Health Organization Quality of Life Assessment. **Bold** indicates *P*<.05.

**Table S3**. Differences in outcome measures from pre-intervention to post-intervention and post-intervention to follow-up for the control group.

| **Measure** | **Pre-intervention (n=26) to post-intervention (n=22)** | | | | **Post-intervention (n=22) to follow-up (n=19)** | | | |
| --- | --- | --- | --- | --- | --- | --- | --- | --- |
|  | Baseline | Baseline +  1 month | Difference | T-test | Baseline +  1 month | Baseline +  3 months | Difference | T-test |
|  | Mean (SD) | Mean (SD) | Mean (95% CI) | *t*(*df*), *P* value | Mean (SD) | Mean (SD) | Mean (95% CI) | *t*(*df*), *P* value |
| Mental health distress (GHQ-12) | 7.54 (3.79) | 3.86 (3.56) | 3.67 (1.53, 5.82) | **3.44(46), .001** | 3.86 (3.56) | 5.26 (4.41) | -1.40 (-3.92, 1.12) | -1.12(39), .27 |
| PTSD symptoms (PCL-C) | 46.58 (14.50) | 39.64 (13.19) | 6.94 (-1.18, 15.06) | 1.72(46), .09 | 39.64 (13.19) | 42.89 (16.29) | -3.26 (-12.57, 6.05) | -0.71(39), .48 |
| Well-being (WEMWBS) | 37.12 (8.98) | 38.68 (9.87) | -1.57 (-7.05, 3.91) | -0.58(46), .57 | 38.68 (9.87) | 39.00 (8.32) | -0.32 (-6.14, 5.50) | -0.11(39), .91 |
| Quality of life (WHOQOL-BREF)  Environment | 13.44 (2.48) | 13.05 (2.91) | 0.40 (-1.17, 1.96) | 0.51(46), .61 | 13.05 (2.91) | 12.39 (2.54) | 0.65 (-1.09, 2.39) | 0.76(39), .45 |
| Physical health | 13.54 (3.16) | 13.53 (2.61) | 0.01 (-1.70, 1.71) | 0.01(46), .99 | 13.53 (2.61) | 13.14 (3.12) | 0.39 (-1.42, 2.20) | 0.44(39), .67 |
| Psychological | 11.28 (2.84) | 10.91 (2.78) | 0.37 (-1.27, 2.01) | 0.46(46), .65 | 10.91 (2.78) | 11.33 (2.99) | -0.42 (-2.25, 1.40) | -0.47(39), .64 |
| Social | 11.44 (4.01) | 11.76 (3.55) | -0.32 (-2.54, 1.90) | -0.29(46), .77 | 11.76 (3.55) | 10.95 (3.81) | 0.81 (-1.52, 3.14) | 0.70(39), .49 |

GHQ-12, General Health Questionnaire; PCL-C, PTSD Checklist Civilian version; WEMBS, Warwick Edinburgh Mental Well-being Scale; WHOQOL-BREF World Health Organization Quality of Life Assessment. **Bold** indicates *P*<.05.

**Table S4**. Differences in outcome measures from pre-intervention to follow-up for the intervention group.

| **Measure** | **Pre-intervention (n=24) to follow-up (n=15)** | | | |
| --- | --- | --- | --- | --- |
|  | Baseline | Baseline +  3 months | Difference | T-test |
|  | Mean (SD) | Mean (SD) | Mean (95% CI) | *t*(*df*), *P* value |
| Mental health distress (GHQ-12) | 6.96 (3.64) | 2.67 (3.60) | 4.29 (1.87, 6.71) | **3.60(37), <.001** |
| PTSD symptoms (PCL-C) | 44.17 (14.95) | 33.2 (13.94) | 10.97 (1.25, 20.69) | **2.29(37), .03** |
| Well-being (WEMWBS) | 38.25 (9.15) | 44.87 (9.36) | -6.62 (-12.77, -0.46) | **-2.18(37), .04** |
| Quality of life (WHOQOL-BREF)  Environment | 12.98 (2.40) | 14.03 (2.43) | -1.05 (-2.66, 0.56) | -1.33(37), .19 |
| Physical health | 13.05 (2.01) | 13.87 (3.41) | -0.82 (-2.57, 0.93) | -0.95(37), .35 |
| Psychological | 11.11 (2.60) | 13.33 (2.40) | -2.22 (-3.91, -0.54) | **-2.67(37), .01** |
| Social | 10.89 (3.60) | 13.24 (3.47) | -2.36 (-4.72, 0.01) | -2.02(37), .05 |

GHQ-12, General Health Questionnaire; PCL-C, PTSD Checklist Civilian version; WEMWBS, Warwick Edinburgh Mental Well-being Scale; WHOQOL-BREF World Health Organization Quality of Life Assessment. **Bold** indicates *P*<.05.

**Table S5**. Differences in outcome measures from pre-intervention to follow-up for the control group.

| **Measure** | **Pre-intervention (n=26) to follow-up (n=19)** | | | |
| --- | --- | --- | --- | --- |
|  | Baseline | Baseline +  3 months | Difference | T-test |
|  | Mean (SD) | Mean (SD) | Mean (95% CI) | *t*(*df*), *P* value |
| Mental health distress (GHQ-12) | 7.54 (3.79) | 5.26 (4.41) | 2.28 (-0.19, 4.75) | 1.86(43), .07 |
| PTSD symptoms (PCL-C) | 46.58 (14.50) | 42.89 (16.29) | 3.68 (-5.62, 12.98) | 0.80(43), .43 |
| Well-being (WEMWBS) | 37.12 (8.98) | 39.00 (8.32) | -1.88 (-7.19, 3.42) | -0.72(43), .48 |
| Quality of life (WHOQOL-BREF)  Environment | 13.44 (2.48) | 12.39 (2.54) | 1.05 (-0.48, 2.57) | 1.39(43), .17 |
| Physical health | 13.54 (3.16) | 13.14 (3.12) | 0.40 (-1.52, 2.31) | 0.42(43), .68 |
| Psychological | 11.28 (2.84) | 11.33 (2.99) | -0.05 (-1.82, 1.72) | -0.06(43), .95 |
| Social | 11.44 (4.01) | 10.95 (3.81) | 0.49 (-1.90, 2.88) | 0.41(43), .68 |

GHQ-12, General Health Questionnaire; PCL-C, PTSD Checklist Civilian version; WEMWBS, Warwick Edinburgh Mental Well-being Scale; WHOQOL-BREF World Health Organization Quality of Life Assessment.
